# Benzene Exposure Alters Expression of Enzymes Involved in Fatty Acid β-Oxidation in Male C3H/He Mice

**DOI:** 10.3390/ijerph13111068

**Published:** 2016-10-31

**Authors:** Rongli Sun, Meng Cao, Juan Zhang, Wenwen Yang, Haiyan Wei, Xing Meng, Lihong Yin, Yuepu Pu

**Affiliations:** Key Laboratory of Environmental Medicine Engineering of Ministry of Education, School of Public Health, Southeast University, Nanjing 210009, Jiangsu, China; sunrongli20609@163.com (R.S.); caomeng8510693@163.com (M.C.); ywwseu@163.com (W.Y.); why_314614@163.com (H.W.); dzmx925@126.com (X.M.); lhyin@seu.edu.cn (L.Y.)

**Keywords:** benzene, fatty acid β-oxidation, mitochondrial dysfunction, oxidative stress

## Abstract

Benzene is a well-known hematotoxic carcinogen that can cause leukemia and a variety of blood disorders. Our previous study indicated that benzene disturbs levels of metabolites in the fatty acid β-oxidation (FAO) pathway, which is crucial for the maintenance and function of hematopoietic and leukemic cells. The present research aims to investigate the effects of benzene on changes in the expression of key enzymes in the FAO pathway in male C3H/He mice. Results showed that benzene exposure caused reduced peripheral white blood cell (WBC), red blood cell (RBC), platelet (Pit) counts, and hemoglobin (Hgb) concentration. Investigation of the effects of benzene on the expression of FA transport- and β-oxidation-related enzymes showed that expression of proteins Cpt1a, Crat, Acaa2, Aldh1l2, Acadvl, Crot, Echs1, and Hadha was significantly increased. The ATP levels and mitochondrial membrane potential decreased in mice exposed to benzene. Meanwhile, reactive oxygen species (ROS), hydrogen peroxide (H_2_O_2_), and malondialdehyde (MDA) levels were significantly increased in the benzene group. Our results indicate that benzene induces increased expression of FA transport and β-oxidation enzymes, mitochondrial dysfunction, and oxidative stress, which may play a role in benzene-induced hematotoxicity.

## 1. Introduction

Benzene is a ubiquitous environmental and occupational pollutant. It is also a recognized cause of acute myeloid leukemia and acute non-lymphocytic leukemia [1,2,3]. Studies showed that benzene causes hematotoxic effects, even at exposure levels lower than 1 ppm [4,5,6]. Benzene metabolites interact with hematopoietic cells at different differentiation stages in bone marrow (BM) [7], resulting in genetic [8], chromosomal, or epigenetic abnormalities [9,10], genomic instability [11], and altered proliferation and differentiation of hematopoietic stem cells (HSCs) [12], leading to the formation of mutated hematopoietic cells and subsequent clonal evolution to leukemia [13]. Although many epidemiological and experimental studies have been carried out, the mechanisms of benzene-induced hematotoxicity remain to be elucidated.

Recent studies demonstrated that fatty acid β-oxidation (FAO) is a crucial pathway for HSCs and leukemic cells. The tight regulation of asymmetric division of HSCs accompanies metabolic changes [14]. Ito et al. reported that inhibition of FAO results in the symmetric commitment of HSC daughter cells, suggesting that FAO functions to control HSC self-renewal [15]. In addition, it was found that the FAO pathway is pivotal for energy production, cell growth, and chemo-resistance in leukemic cells [16,17]. The results of Ricciardi and colleagues suggest that FAO can be a potential target for leukemia treatment [16]. Our previous study indicated that benzene exposure induces disturbances in the metabolite levels of the FAO pathway [18]. Therefore, FAO may be a key pathway related to benzene-induced hematotoxicity. However, specific effects of benzene on enzymes involved in the FAO pathway remain unclear.

In the present study, we examine the effects of benzene on the expression of key enzymes in the FAO pathway, as well as its effects on mitochondrial function and oxidative stress. The results may be helpful in further understanding the mechanism of action for the hematotoxicity of benzene.

## 2. Materials and Methods

### 2.1. Chemicals and Antibodies

Benzene was obtained from Sigma (St. Louis, MO, USA). The β-actin antibody was purchased from Santa Cruz (Santa Cruz, CA, USA), all other primary antibodies (carnitine palmitoyltransferase 1a (Cpt1a), carnitine acetyltransferase (Crat), acetyl-coenzyme A acyltransferase 2 (Acaa2), aldehyde dehydrogenase 1 family, member L2 (Aldh1l2), very long chain acyl-coenzyme A dehydrogenase (Acadvl), carnitine O-octanoyltransferase (Crot), enoyl coenzyme A hydratase short chain 1 (Echs1), and hydroxyacyl-coenzyme A dehydrogenase/3-ketoacyl-coenzyme A thiolase/enoyl-coenzyme A hydratase, alpha subunit (Hadha) were obtained from Abcam (Cambridge, MA, USA).

### 2.2. Animals and Treatment

Male C3H/He mice aged 4–5 weeks were purchased from Wei Tong Li Hua Laboratory Animal Co. Ltd. (Beijing, China). Mice were maintained at 22–24 °C with 45%–65% humidity and a 12-h alternating light/dark cycle. Standard diet and water were available ad libitum. After one week of acclimatization, the mice were divided into two groups (eight per group): control, and mice treated with 150 mg/kg benzene. There are three reasons for the choice of this benzene exposure dose. First, normal occupational exposures vary from less than 1 ppm to hundreds of ppm, the latter resulted in decreased numbers of white blood cells (WBC), red blood cells (RBC), and/or platelets (Pit) in the peripheral blood of workers [5,13]; second, the dose of 150 mg/kg body weight (b.w.) benzene is equivalent to the inhalation protocol with 300 ppm [19]. It is reported that a dose of 300 ppm benzene inhalation induced 9% acute myeloid leukemias (AMLs) in wild-type C3H/He mice [20]; third, based on our previous study, significant hematotoxicity was observed in C3H/He mice exposed to 150 mg/kg b.w. for four weeks. Mice were injected subcutaneously with either corn oil or a benzene–corn oil mixture once daily, five days per week, for four weeks. Animals were maintained and experiments were conducted in accordance with the Institutional Animal Care and Use Committee of Southeast University (Approval No: 20130027).

### 2.3. Blood Routine

The mouse body weight was measured every week. At the end of four weeks, 200 μL of blood was collected from mouse orbital sinus into an ethylene diamine tetraacetic acid (EDTA)-treated tube. The blood parameters were measured using a Sysmex XE-2100 fully automatic hematology analyzer (Sysmex, Kobe, Japan).

### 2.4. L-Carnitine Assay

The BM cells were acquired from mouse femurs and tibias. Both ends of the bones were cut, and marrow cavities were washed with buffer. Mouse BM cells were centrifuged at 1000 rpm for 10 min at 4 °C. Then, an l-carnitine assay kit from Sigma (St. Louis, MO, USA) was used to measure l-carnitine in BM cells. Briefly, 1 × 10^6^ cells were homogenized in 100 μL carnitine assay buffer and centrifuged at 13,000 rpm for 10 min to remove insoluble material. Sample (50 μL) was mixed with 50 μL of reaction mix by pipetting in a 96-well plate. The reaction was incubated for 30 min at room temperature in the dark. Finally, the absorbance was measured at 570 nm.

### 2.5. Real-Time Polymerase Chain Reaction (PCR)

The BM cells were collected, and total RNA was extracted using TRIzol (Invitrogen, New York, NY, USA). The cDNA was synthesized using PrimeScriptTM RT Master Mix (Takara, Otsu, Japan), according to the manufacturer’s instructions. Real-time PCR was performed using SYBR Green Realtime PCR Master Mix (Toyobo, Osaka, Japan). All the samples were analyzed in triplicate. The relative gene expression was analyzed by the 2^−∆∆Ct^ method. Actin was used as the reference gene. The following primer sequences were used for PCR (Table 1).

### 2.6. Western Blot

The mouse BM cells were harvested and lysed in 100 μL radio-immunoprecipitation assay (RIPA) buffer with 1 mM phenylmethylsulfonyl fluoride (PMSF). The protein concentration was measured by using Pierce bicinchoninic acid (BCA) protein assay kit (Thermo Scientific, Hudson, NH, USA). Proteins (20 μg) were separated by sodium dodecyl sulfate polyacrylamide gel electrophoresis (SDS-PAGE) with 10% separation gel and 5% space gel. Then, proteins were transferred to a polyvinylidene difluoride (PVDF) membrane and incubated with 5% nonfat milk for 1 h at room temperature. Primary antibodies were optimized and incubated with membrane overnight at 4 °C. The next day, the membrane was washed with tris-buffered saline with Tween 20 (TBST) buffer and incubated with secondary antibody for 1 h at room temperature. Signals were visualized and analyzed using a chemiluminescent substrate (Thermo Scientific, Hudson, NH, USA).

### 2.7. ATP Assay

Intracellular ATP levels were assayed by a firefly luciferase-based ATP assay kit (Beyotime, Nantong, China) according to the manufacturer’s instructions. Briefly, 1 × 10^6^ cells were lysed with 100 μL lysis buffer and centrifuged at 12,000 rpm for 5 min at 4 °C. Then, 20 μL of each supernatant were mixed with 100 μL of ATP detection working solution and assayed by a luminometer (Berthold Detection System, Pforzheim, Germany).

### 2.8. Mitochondrial Membrane Potential Measurement

JC-1 dye was used to measure membrane potential. The BM cells were collected and incubated with JC-1 for 20 min at 37 °C. The fluorescence intensity was detected with a BD fluorescence activating cell sorter (FACS) Aria II flow cytometry unit (BD Bioscience, San Diego, CA, USA). The ratio of red/green fluorescence was used to indicate the mitochondrial membrane potential.

### 2.9. Measurement of Reactive Oxygen Species (ROS) Level

The intracellular ROS was detected using oxidant-sensitive dye DCFH-DA (Beyotime, Nantong, China). The cells were incubated with 2 mL 10 μM DCFH-DA working solution at 37 °C for 20 min. Then, the cells were washed twice, and the fluorescence was determined by flow cytometry.

### 2.10. Measurement of H_2_O_2_ Level

H_2_O_2_ concentration was determined using H_2_O_2_ assay kit (Beyotime, Nantong, China). The cell lysate reacted with working solution and the formed complex was measured using a microplate reader at a wavelength of 560 nm.

### 2.11. Measurement of Malondialdehyde (MDA) Level

The cellular MDA level was measured using a Lipid Peroxidation MDA Assay Kit (Beyotime, Nantong, China). The cells were lysed, and supernatant was reacted with thiobarbituric acid (TBA) to generate the MDA-TBA adduct. Then, the products were measured spectrophotometrically at 535 nm, and the MDA levels were expressed as μmol/mg protein.

### 2.12. Statistical Analysis

The results are expressed as mean ± SD. Statistically significant differences between groups were determined using one-way ANOVA, followed by Dunnett’s multiple comparison tests. A *p*-value < 0.05 was considered as statistically significant.

## 3. Results

### 3.1. Effects of Benzene on Body Weight

**Figure 1:**
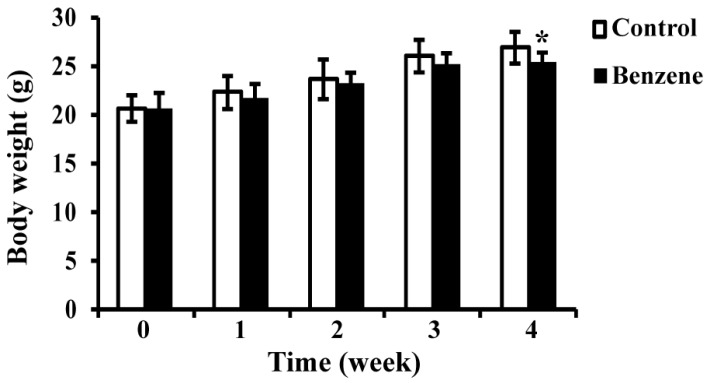
Effects of benzene exposure on body weight (g). Data are represented as the Mean ± SD (*n* = 8). * *p* < 0.05 compared with control group.

We measured body weights of mice in two groups over the four-week exposure period; the results are shown in Figure 1. Benzene exposure caused a decrease in mouse body weight compared with control, but a significant difference was only seen at the fourth week.

### 3.2. Benzene Causes Declines in Mouse WBC, RBC, Pit Count, and Hgb Concentration

We then evaluated the effects of benzene exposure on blood routine. As shown in Table 2, mice in the benzene exposure group displayed a significant decrease in the number of WBC, RBC, Pit, and a decline in Hgb concentration.

### 3.3. Benzene Induces Reduced L-Carnitine and Alters Expression of Key Genes in the FAO Pathway in Mouse BM Cells

**Figure 2:**
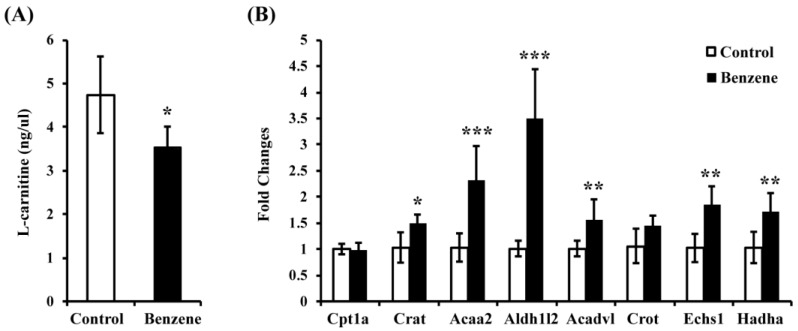
Benzene induces reduced l-carnitine and alters expression of key genes in the fatty acid β-oxidation (FAO) pathway in mouse bone marrow (BM) cells. (**A**) l-carnitine level in control and benzene group; (**B**) FAO-related gene expression in two groups. Values are mean ± SD; Compared with control group: * *p* < 0.05; ** *p* < 0.01; *** *p* < 0.001. Cpt1a: carnitine palmitoyltransferase 1a; Crat: carnitine acetyltransferase; Acaa2: acetyl-coenzyme A acyltransferase 2; Aldh1l2: aldehyde dehydrogenase 1 family, member L2; Acadvl: very long chain acyl-coenzyme A dehydrogenase; Crot: carnitine O-octanoyltransferase; Echs1: enoyl coenzyme A hydratase short chain 1; Hadha: hydroxyacyl-coenzyme A dehydrogenase/3-ketoacyl-coenzyme A thiolase/enoyl-coenzyme A hydratase, alpha subunit.

We previously reported that acetyl-l-carnitine (ALCAR) level decreased in mouse BM cells due to benzene exposure. Here, we also found significantly reduced l-carnitine level in the benzene group (Figure 2A). These results indicate that benzene exposure induced disturbances in the metabolite levels of the FAO pathway. We were then interested in whether benzene exposure affects expression of key genes in the FAO pathway. To investigate this, we performed real-time (RT)-PCR to detect gene expressions of Cpt1a, Crat, Acaa2, Aldh1l2, Acadvl, Crot, Echs1, and Hadha. As shown in Figure 2B, the mRNA levels of Crat, Acaa2, Aldh1l2, Acadvl, Echs1, and Hadha were significantly increased in the benzene group, while no significant difference was found in the mRNA expression of Cpt1a and Crot in the benzene group.

### 3.4. Benzene Induces Increased Expression of Key Fatty Acid (FA) Transport Proteins in Mouse BM Cells

**Figure 3:**
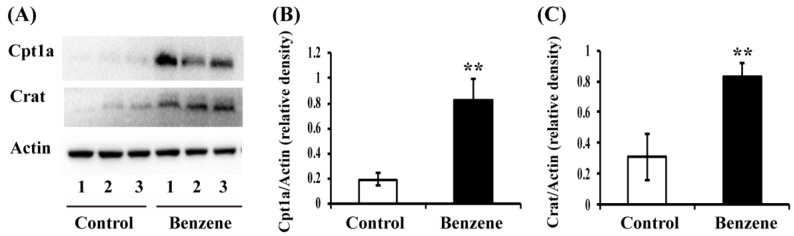
Benzene induces increased expression of key fatty acid (FA) transport proteins in mouse BM cells. Male C3H/He mice were exposed to benzene for 4 weeks, and BM cells were collected. Then, Cpt1a and Crat protein levels were detected by Western blot (**A**). The relative expression of Cpt1a (**B**) and Crat (**C**) was calculated by densitometric scanning of band intensity. Values are mean ± SD; ** *p* < 0.01, compared with control group.

L-carnitine and ALCAR are important intermediates in FAO, and play a vital role in carrying FA across the inner mitochondrial membrane. Cpt1a and Crat are two key enzymes involved in FA transport. To test whether benzene exposure affects ALCAR-related FA transport proteins, Cpt1a and Crat protein expression was evaluated by Western blot. As shown in Figure 3, the protein levels of Cpt1a (*p* < 0.01) and Crat (*p* < 0.01) were significantly higher in BM cells of benzene-exposed mice than that in the control group.

### 3.5. Benzene Induces Increased Expression of Key FAO Proteins in Mouse BM Cells

**Figure 4:**
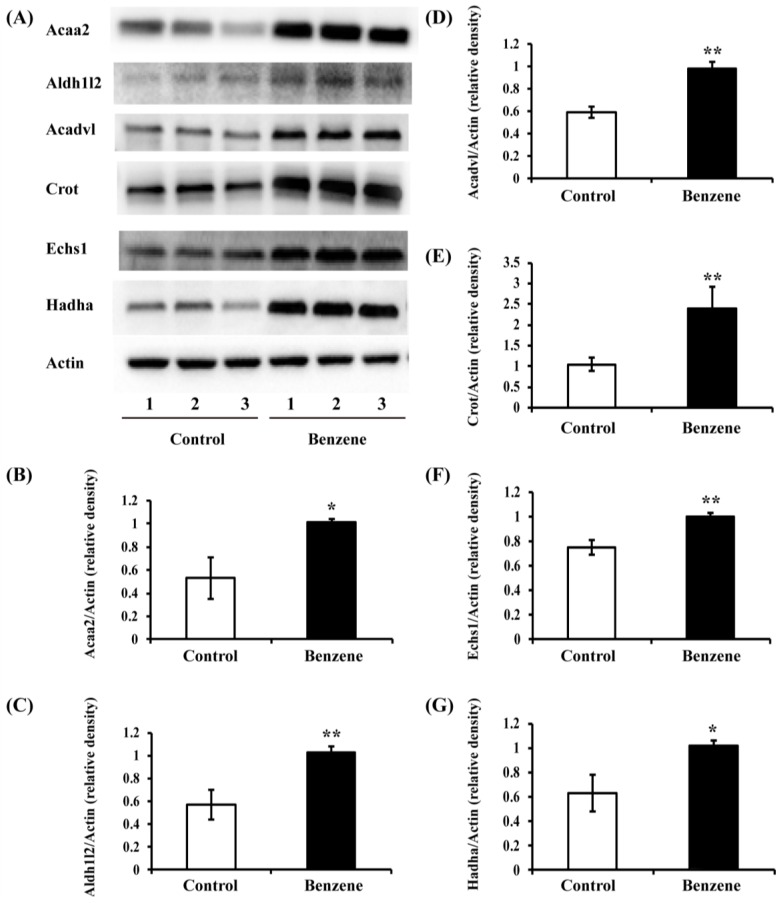
Benzene induces increased expression of key FAO proteins in mouse BM cells. Male C3H/He mice were exposed to benzene for 4 weeks, and BM cells were collected. Then, Acaa2, Aldh1l2, Acadvl, Crot, Echs1, and Hadha protein levels were detected by Western blot (**A**). The relative expression of Acaa2 (**B**); Aldh1l2 (**C**); Acadvl (**D**); Crot (**E**); Echs1 (**F**); and Hadha (**G**) was calculated by densitometric scanning of band intensity. Values are mean ± SD; compared with control group: * *p* < 0.05; ** *p* < 0.01.

Acaa2, Aldh1l2, Acadvl, Crot, Echs1, and Hadha are key enzymes in the FAO pathway. The effects of benzene on these enzymes in mRNA level are shown in Figure 2. Meanwhile, we were also interested in whether benzene exposure affects protein expression of these enzymes. To investigate this, we performed Western blot analysis. The results showed that the protein levels of Acaa2, Aldh1l2, Acadvl, Crot, Echs1, and Hadha increased significantly in mice exposed to benzene (Figure 4).

### 3.6. Benzene Induces Mitochondrial Dysfunction

**Figure 5:**
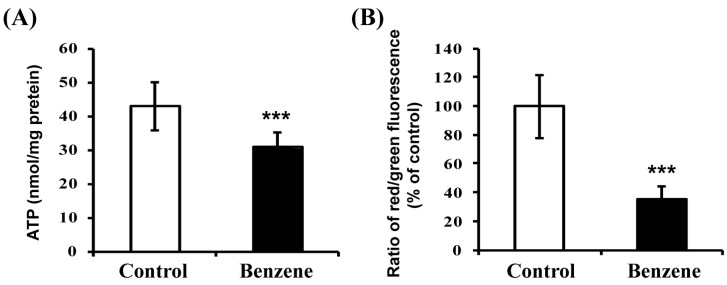
Benzene causes mitochondrial dysfunction in mouse BM cells. The ATP level (**A**) and mitochondrial membrane potential (**B**) were determined. Values are mean ± SD; *** *p* < 0.001, compared with control group.

Because FAO occurs mainly in mitochondria, we next measured ATP level and mitochondrial membrane potential in BM cells to evaluate mitochondrial function (Figure 5). Both ATP level and ratio of red/green florescence showed a significant reduction in the benzene group, indicating that benzene causes mitochondrial damage.

### 3.7. Benzene Induces Oxidative Stress and Lipid Peroxidation

**Figure 6:**
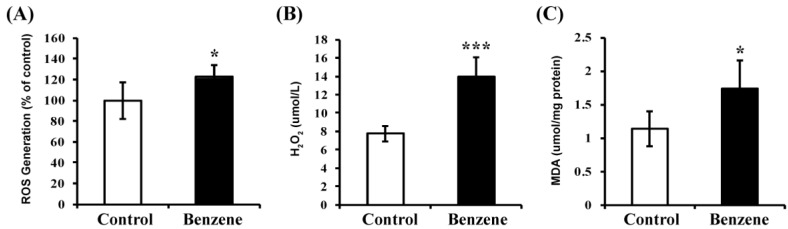
Benzene causes oxidative stress and lipid peroxidation in mouse BM cells. The oxidative stress was evaluated by measuring reactive oxygen species (ROS) production (**A**) and H_2_O_2_ level (**B**). The lipid peroxidation was determined by assessing malondialdehyde (MDA) level (**C**). Values are mean ± SD; compared with control group: * *p* < 0.05; *** *p* < 0.001.

Since FAO is an important source of NADPH, which is a key member of the antioxidant family, we then evaluated the ROS and H_2_O_2_ levels in BM cells of benzene-exposed mice. The results showed that significantly elevated ROS and H_2_O_2_ levels were detected in benzene groups (Figure 6). Then, the levels of MDA (an end product of lipid peroxidation) were measured to determine the impact of benzene on oxidative lesions. MDA concentration was significantly increased in benzene groups (*p* < 0.05) (Figure 6). These results indicated that benzene induces oxidative stress and lipid peroxidation in BM cells of C3H/He mice.

## 4. Discussion

Benzene is classified as a carcinogen [21], as benzene exposure causes leukemia and other hematopoietic disorders [2,5]. However, the mechanism of hematotoxicity induced by benzene has not been well understood so far. Our previous metabonomics study [18] suggested that benzene exposure causes changes in FAO-related metabolites in BM cells in C3H/He mice. In the present study, we investigated the effects of benzene on the expression of FAO-related enzymes in C3H/He mice.

We first examined the complete blood count to evaluate benzene-induced hematotoxicity. Consistent with previous studies, a decreased number of WBC, RBC, Pit, and Hgb concentration were found in benzene group, which was considered to be the result of aplastic anemia [5]. This result indicated that significant hematotoxicity was successfully induced by benzene exposure in our mouse model. The cellularity changes in specific lineages after benzene exposure were not investigated here. In our previous study [22,23], the dose of benzene used could also cause significantly decreased frequency of HSCs (Lin^−^ C-kit^+^ Sca-1^+^), increased ratio of original granulocyte and promyelocytes in bone marrow, and reduced burst-forming unit-erythroid (BFU-E), committed progenitors granulocytes-erythroid-monocyte-megakaryocyte (CFU-GEMM), and granulocyte- macrophages (CFU-GM) progenitors [23].

FAO plays a pivotal role in energy homoeostasis. Carnitine and ALCAR are essential for the FAO pathway due to their role in FA transport into the mitochondrial matrix via the carnitine/ALCAR shuttle. Studies showed that carnitine and ALCAR levels are associated with type 2 diabetes and insulin resistance, suggesting that FAO plays a role in the pathogenesis of diabetes [24,25]. Claudia et al. reported that drug-resistant human T-lymphoblastic leukemia (CEM) cells exhibited reduced levels of short-chain acyl carnitines, implying a reduction in the FAO rate [26]. Here, we found decreased levels of l-carnitine and ALCAR in BM cells of mice exposed to benzene. Based on this, we hypothesized that benzene exposure would affect the FAO process, which may be involved in the pathogenesis of benzene-induced hematotoxicity.

Previous research indicated that FAO is a key metabolic pathway for hematopoietic and leukemic cell maintenance and function [27]. It was reported that the promyelocytic leukemia (PML)-peroxisome proliferator-activated receptor δ (PPAR-δ)-FAO pathway controls HSC cell fate through the regulation of HSC asymmetric division [15]. Ismael et al. found that the inhibition of FAO significantly decreased the number of quiescent leukemia progenitor cells in half of primary human acute myeloid leukemia samples [17]. Fatty acids are transported from cytosol to mitochondria via the carnitine shuttle, then undergo β-oxidation. Cpt1a and Crat are two key enzymes involved in the entry of FA into the mitochondria. Results reported by Maria et al. demonstrated that leukemia cell lines constitutively express Cpt1a [16]. Our results showed that Cpt1a protein, but not mRNA, was increased in BM cells of mouse. Benzene exposure significantly increased mRNA and protein levels of Crat in mouse BM cells. Therefore, FA transport may be affected due to benzene exposure.

Once inside the mitochondria, acyl-CoAs are metabolized and shortened by multiple enzymes, including Acaa2, Aldh1l2, Acadvl, Crot, Echs1, and Hadha. Acaa2 was reported to be differentially expressed in CEM and drug-resistant leukemia cells [26]. Crot expression was found to be over-expressed in multidrug-resistant K562 leukemia cells [28]. In the current study, we found mRNA and protein levels of Acaa2, Aldh1l2, Acadvl, Echs1, and Hadha were significantly increased due to benzene exposure. Crot was only significantly increased in protein level after benzene exposure. These results indicate that benzene exposure results in abnormal FA transport and FAO process in mouse BM cells, which may play a role in hematotoxicity induced by benzene.

FAO is carried out in mitochondria, which are the primary source and target of ROS and lipid peroxidation [29]. Under normal circumstances, the vast network of antioxidant and detoxification systems ensures that levels of ROS and lipid peroxidation are kept at sub-toxic levels [29]. The FAO process sustains ATP levels and NADPH production [30,31]. Increased ROS could lead to the exhaustion of HSCs [32]. FAO could provide stem cells with the NADPH to prevent ROS from driving cell differentiation [27]. So, FAO may play a role in the mitochondrial antioxidant system. Our results showed that benzene induces significantly-reduced ATP levels and mitochondrial membrane potential, indicating mitochondrial dysfunction. In addition, benzene exposure also results in oxidative stress by generating increased ROS and H_2_O_2_, and leads to increased lipid peroxidation. Lipid peroxidation products such as MDA eventually damage the hematopoietic cells at different stages of differentiation [33]. Thus, aberrant FAO that produces less ATP and NADPH may be associated with mitochondrial dysfunction and oxidative stress, and may participate in hematotoxicity induced by benzene.

## 5. Conclusions

In conclusion, this is the first report to demonstrate that benzene exposure causes alterations in the expression of key enzymes involved in the FAO pathway in mouse BM cells. We also found that benzene exposure induces mitochondrial dysfunction, oxidative stress, and lipid peroxidation. Further studies are needed to explore the definite mechanism responsible for benzene-induced aberrant FAO and to better understand the role of abnormal FAO in the pathogenesis of benzene-induced hematotoxicity.

## Figures and Tables

**Table 1:** Real-time polymerase chain reaction (PCR) primers used in this study.

Primer Name	Primer Sequence (5′-3′)
Actin	Forward: CGAGCGGTTCCGATGCCCTG; Reverse: ACGCAGCTCAGTAACAGTCCGC
Cpt1a	Forward: CGATCATCATGACTATGCGCTACT; Reverse: GCCGTGCTCTGCAAACATC
Crat	Forward: GCTTGTGCGGTCTCCTATGGT; Reverse: TTGGCTTTCTCTATGTCGTTCTTG
Hadha	Forward: AAGGGGATGTGGCAGTTATT; Reverse: ACTCCTGATTTGGTCGTTGG
Acaa2	Forward: CAATGAGGAGATGGCACCC; Reverse: CTTTCTTGAACACGGACGG
Acadvl	Forward: GTGGCTCTGCAAGGCTGTA; Reverse: CGATTCCTGTCCTCCGTCTC
Echs1	Forward: AAGCAGGCAGGTCTTGTAAGC; Reverse: CGGTGGCAAAGGTGGAATA
Aldh1l2	Forward: ACCAGCCGGGTTTATTTCAAA; Reverse: ACTCCCACTACTCGGTGGC
Crot	Forward: GGTGGCTCAATGTTGCCTAC; Reverse: TCCTCTTTCTAACTGAGTGCCT

**Table 2:** Effects of benzene on blood routine. WBC: white blood cell; RBC: red blood cell; Hgb: hemoglobin; Pit: platelet.

Group	WBC (10^9^/L)	RBC (10^12^/L)	Hgb (g/L)	Pit (10^9^/L)
Control	7.36 ± 1.45	8.04 ± 0.18	127.75 ± 11.32	593.38 ± 87.43
Benzene	3.08 ± 0.80 ***	6.23 ± 0.33 *	109.50 ± 5.50 *	368.38 ± 82.63 *

Note. * *p* < 0.05 compared with control group; *** *p* < 0.001 compared with control group.

## References

[1] Infante P.F. (2011). The IARC October 2009 evaluation of benzene carcinogenicity was incomplete and needs to be reconsidered. Am. J. Ind. Med..

[2] Lan Q., Zhang L., Li G., Vermeulen R., Weinberg R.S., Dosemeci M., Rappaport S.M., Shen M., Alter B.P., Wu Y. (2004). Hematotoxicity in workers exposed to low levels of benzene. Science.

[3] Carbonari D., Chiarella P., Mansi A., Pigini D., Iavicoli S., Tranfo G. (2016). Biomarkers of susceptibility following benzene exposure: Influence of genetic polymorphisms on benzene metabolism and health effects. Biomark. Med..

[4] Swaen G.M., van Amelsvoort L., Twisk J.J., Verstraeten E., Slootweg R., Collins J.J., Burns C.J. (2010). Low level occupational benzene exposure and hematological parameters. Chem. Biol. Interact..

[5] Snyder R. (2012). Leukemia and benzene. Int. J. Environ. Res. Public Health.

[6] Schnatter A.R., Glass D.C., Tang G., Irons R.D., Rushton L. (2012). Myelodysplastic syndrome and benzene exposure among petroleum workers: An international pooled analysis. J. Natl. Cancer Inst..

[7] Ross D. (2000). The role of metabolism and specific metabolites in benzene-induced toxicity: Evidence and issues. J. Toxicol. Environ. Health A.

[8] Gao A., Yang J., Yang G., Niu P., Tian L. (2014). Differential gene expression profiling analysis in workers occupationally exposed to benzene. Sci. Total Environ..

[9] Zhang L., Rothman N., Li G., Guo W., Yang W., Hubbard A.E., Hayes R.B., Yin S., Lu W., Smith M.T. (2007). Aberrations in chromosomes associated with lymphoma and therapy-related leukemia in benzene-exposed workers. Environ. Mol. Mutagen..

[10] Xing C., Wang Q.F., Li B., Tian H., Ni Y., Yin S., Li G. (2010). Methylation and expression analysis of tumor suppressor genes p15 and p16 in benzene poisoning. Chem. Biol. Interact..

[11] Gowans I.D., Lorimore S.A., McIlrath J.M., Wright E.G. (2005). Genotype-dependent induction of transmissible chromosomal instability by gamma-radiation and the benzene metabolite hydroquinone. Cancer Res..

[12] Zhou H., Dehn D., Kepa J.K., Siegel D., Scott D.E., Tan W., Ross D. (2010). NAD(P)H:quinone oxidoreductase 1-compromised human bone marrow endothelial cells exhibit decreased adhesion molecule expression and CD34+ hematopoietic cell adhesion. J. Pharmacol. Exp. Ther..

[13] McHale C.M., Zhang L., Smith M.T. (2012). Current understanding of the mechanism of benzene-induced leukemia in humans: Implications for risk assessment. Carcinogenesis.

[14] Ito K., Suda T. (2014). Metabolic requirements for the maintenance of self-renewing stem cells. Nat. Rev. Mol. Cell Biol..

[15] Ito K., Carracedo A., Weiss D., Arai F., Ala U., Avigan D.E., Schafer Z.T., Evans R.M., Suda T., Lee C.H. (2012). A PML-PPAR-delta pathway for fatty acid oxidation regulates hematopoietic stem cell maintenance. Nat. Med..

[16] Ricciardi M.R., Mirabilii S. (2015). Targeting the leukemia cell metabolism by the CPT1a inhibition: Functional preclinical effects in leukemias. Boold.

[17] Samudio I., Harmancey R., Fiegl M., Kantarjian H., Konopleva M., Korchin B., Kaluarachchi K., Bornmann W., Duvvuri S., Taegtmeyer H. (2010). Pharmacologic inhibition of fatty acid oxidation sensitizes human leukemia cells to apoptosis induction. J. Clin. Investig..

[18] Sun R., Zhang J., Yin L., Pu Y. (2014). Investigation into variation of endogenous metabolites in bone marrow cells and plasma in C3H/He mice exposed to benzene. Int. J. Mol. Sci..

[19] Inoue T., Hirabayashi Y. (2010). Hematopoietic neoplastic diseases develop in C3H/He and C57BL/6 mice after benzene exposure: Strain differences in bone marrow tissue responses observed using microarrays. Chem. Biol. Interact..

[20] Kawasaki Y., Hirabayashi Y., Kaneko T., Kanno J., Kodama Y., Matsushima Y., Ogawa Y., Saitoh M., Sekita K., Uchida O. (2009). Benzene-induced hematopoietic neoplasms including myeloid leukemia in Trp53-deficient C57BL/6 and C3H/He mice. Toxicol. Sci..

[21] Cogliano V.J., Baan R., Straif K. (2011). Updating IARC’s carcinogenicity assessment of benzene. Am. J. Ind. Med..

[22] Sun R., Zhang J., Xiong M., Chen Y., Yin L., Pu Y. (2012). Metabonomics biomarkers for subacute toxicity screening for benzene exposure in mice. J. Toxicol. Environ. Health Part A.

[23] Sun R., Zhang J., Xiong M., Wei H., Tan K., Yin L., Pu Y. (2015). Altered expression of genes in signaling pathways regulating proliferation of hematopoietic stem and progenitor cells in mice with subchronic benzene exposure. Int. J. Environ. Res. Public Health.

[24] Houten S.M., Wanders R.J. (2010). A general introduction to the biochemistry of mitochondrial fatty acid beta-oxidation. J. Inherit. Metab. Dis..

[25] Mihalik S.J., Goodpaster B.H., Kelley D.E., Chace D.H., Vockley J., Toledo F.G., DeLany J.P. (2010). Increased levels of plasma acylcarnitines in obesity and type 2 diabetes and identification of a marker of glucolipotoxicity. Obesity.

[26] Staubert C., Bhuiyan H., Lindahl A., Broom O.J., Zhu Y., Islam S., Linnarsson S., Lehtio J., Nordstrom A. (2015). Rewired metabolism in drug-resistant leukemia cells: A metabolic switch hallmarked by reduced dependence on exogenous glutamine. J. Biol. Chem..

[27] Carracedo A., Cantley L.C., Pandolfi P.P. (2013). Cancer metabolism: Fatty acid oxidation in the limelight. Nat. Rev. Cancer.

[28] Lehne G., Grasmo-Wendler U.H., Berner J.M., Meza-Zepeda L.A., Adamsen B.L., Flack A., Reiner A., Clausen O.P., Hovig E., Myklebost O. (2009). Upregulation of stem cell genes in multidrug resistant K562 leukemia cells. Leuk. Res..

[29] Anderson E.J., Katunga L.A., Willis M.S. (2012). Mitochondria as a source and target of lipid peroxidation products in healthy and diseased heart. Clin. Exp. Pharmacol. Physiol..

[30] Schafer Z.T., Grassian A.R., Song L., Jiang Z., Gerhart-Hines Z., Irie H.Y., Gao S., Puigserver P., Brugge J.S. (2009). Antioxidant and oncogene rescue of metabolic defects caused by loss of matrix attachment. Nature.

[31] Jeon S.M., Chandel N.S., Hay N. (2012). AMPK regulates NADPH homeostasis to promote tumour cell survival during energy stress. Nature.

[32] Hosokawa K., Arai F., Yoshihara H., Nakamura Y., Gomei Y., Iwasaki H., Miyamoto K., Shima H., Ito K., Suda T. (2007). Function of oxidative stress in the regulation of hematopoietic stem cell-niche interaction. Biochem. Biophys. Res. Commun..

[33] Srinivasan P., Sabitha K.E., Shyamaladevi C.S. (2007). Attenuation of 4-nitroquinoline 1-oxide induced in vitro lipid peroxidation by green tea polyphenols. Life Sci..

